# Meta-Analysis on Pharmacogenetics of Platinum-Based Chemotherapy in Non Small Cell Lung Cancer (NSCLC) Patients

**DOI:** 10.1371/journal.pone.0038150

**Published:** 2012-06-26

**Authors:** Ji-Ye Yin, Qiong Huang, Ying-Chun Zhao, Hong-Hao Zhou, Zhao-Qian Liu

**Affiliations:** 1 Institute of Clinical Pharmacology, Hunan Key Laboratory of Pharmacogenetics, Central South University, Changsha, China; 2 Institute of Clinical Pharmacology, Anhui Medical University, Key Laboratory of Anti-Inflammatory and Immunopharmacology of Education Ministry, Hefei, Anhui, China; 3 Osteoporosis Research Center, Creighton University, Omaha, Nebraska, United States of America; H. Lee Moffitt Cancer Center & Research Institute, United States of America

## Abstract

**Aim:**

To determine the pharmacogenetics of platinum-based chemotherapy in Non Small Cell Lung Cancer (NSCLC) patients.

**Methods:**

Publications were selected from PubMed, Cochrane Library and ISI Web of Knowledge. A meta-analysis was conducted to determine the association between genetic polymorphisms and platinum-based chemotherapy by checking odds ratio (OR) and 95% confidence interval (CI).

**Results:**

Data were extracted from 24 publications, which included 11 polymorphisms in 8 genes for meta-analysis. MDR1 C3435T (OR = 1.97, 95% CI: 1.11–3.50, *P* = 0.02), G2677A/T (OR = 2.61, 95% CI: 1.44–4.74, *P* = 0.002) and GSTP1 A313G (OR = 0.32, 95% CI: 0.17–0.58, *P* = 0.0002) were significantly correlated with platinum-based chemotherapy in Asian NSCLC patients.

**Conclusion:**

Attention should be paid to MDR1 C3435T, G2677A/T and GSTP1 A313G for personalized chemotherapy treatment for NSCLC patients in Asian population in the future.

## Introduction

Lung cancer is one of the most serious public health problems around the globe. There are two major forms of lung cancer: small cell lung cancer (SCLC) and non small cell lung cancer (NSCLC), with the latter accounting for 85% of total cases [1]. Although intensive effort has been made to improve the efficacy of lung cancer therapy, the 5-year relative survival rate still remains between 11% and 17% [2].

Platinum-based chemotherapy is one of the major therapeutic methods for NSCLC, especially for advanced cancer. However, in clinical practice, the chemotherapy response varies wildly among individuals. Some patients respond to the chemotherapy, while others confer intrinsic or acquired resistance. It is speculated that some genetic polymorphisms may affect drug response, and there are accumulating evidences to support this hypothesis [3]–[5]. Therefore, understanding the association between the polymorphisms and platinum response will be beneficial for individualized chemotherapy. Although a number of studies investigated this issue, there is no congruency for the relationship between genetic polymorphisms and NSCLC platinum-based chemotherapy response. Results from different studies are inconsistent with one another. For example, Su *et al.* found excision repair cross-complementing 1 (ERCC1) C354T was significantly associated with drug response while other studies presented contradictory results [6]–[8]. The same situation exists for GSTP1 A313G [9]–[11]. A recent review summarized the pharmacogenomics of platinum-based chemotherapy in NSCLC [12]. However, a quantitative evaluation is still lacking.

The aim of this study is to provide a comprehensive assessment on the association between genetic polymorphisms and platinum-based drug response in NSCLC. We collected all available publications on pharmacogenetic studies of platinum-based chemotherapy in NSCLC, and quantitatively studied them using meta-analysis.

## Materials and Methods

Literature search: A systematic literature search was performed independently by two authors (J.Y.Y. and Q.H.) in three electronic databases: PubMed database, Cochrane Library and ISI Web of Knowledge. The identified articles were reviewed carefully to find more relevant articles. All search results were reviewed and compared by a third reviewer (Z.Q.L.) and the discrepancies between searchers were discussed and solved with consensus. The time period for literature searching was from the first available article to April 1^th^ 2012. Publications were retrieved using terms related to platinum drugs (“platinum” or “cisplatin” or “carboplatin” or “oxaliplatin”) in combination with keywords related to genetic variation (“polymorphism” or “SNP” or “single nucleotide polymorphism” or “mutation” or “variation”) and “lung cancer”.

Publication selection criteria: Publications meeting the following criteria were included: (a) patients with NSCLC; (b) trials had platinum-based chemotherapy; (c) the data of response rate stratified by polymorphisms could be obtained or derived from the original article or corresponding author. Studies were excluded if any of the following applies: (a) papers written in a language other than English; (b) the data of response rate stratified by polymorphisms could not be provided; (c) repeated publications, abstracts, letters, or review articles.

Data extraction: Data were manually extracted independently by two authors (J.Y.Y. and Q.H.), who were blind to each other, using the same data recording form. After exaction, all results were reviewed and compared by a third reviewer (Z.Q.L.) and the discrepancies between extractors were discussed and solved with consensus. When necessary, data of some studies were obtained directly from corresponding authors. The following information of each study was collected: first author’s name, publication year, ethnicity (country), sample size, polymorphisms, dbSNP number of investigated polymorphisms, genotyping methods, disease stage, chemotherapeutic drugs, and the number of responders and non-responders in different genotypes.

Data analysis: The patients were divided into two groups: responders (including complete responders (CR) and partial responders (PR)) and non-responders (including stable disease (SD) and progressive disease (PD)). All data were loaded into and analyzed by the Cochrane Collaboration software (Review Manager 5, the Cochrane Collaboration, Oxford, UK). The pooled odds ratio (OR) and associated 95% confidence interval (CI) were calculated. The significance of the pooled OR was determined using the Z test. The heterogeneity of publications in each meta-analysis was evaluated by I^2^ statistical analysis, which describes the proportion of total variation across studies due to heterogeneity rather than chance. As a guide, I^2^ value ranged from 0 to 100%, and values of 25%, 50% and 75% were considered as low, moderate and high heterogeneity respectively [13]. To further evaluate the extent of heterogeneity between publications, Cochran’s χ^2^ based Q statistic test was also employed [14]. The threshold significance level of heterogeneity was set at *P* = 0.10. If *P*<0.10, the random-effect model using DerSimonian and Laird method was selected to pool the results, yielding wider CIs [15], [16]. Otherwise, the pooled ORs and *P* values were calculated by fixed-effect model using the Mantel-Haenszel method [17]. To control publication bias, the relationship between OR and SE (log [OR]) was estimated using funnel plot. The symmetry of funnel plots was visually inspected. To further evaluate publication bias, Begg’s test [18] and Egger’s test [19] were also performed using Stata 12.0 software (StataCorp LP, College Station, USA). A *P*<0.05 was considered statistically significant in all analyses, except for heterogeneity test.

## Results

### 1. Literature Review, Characteristics of Studies and Publication Bias

**Figure 1:**
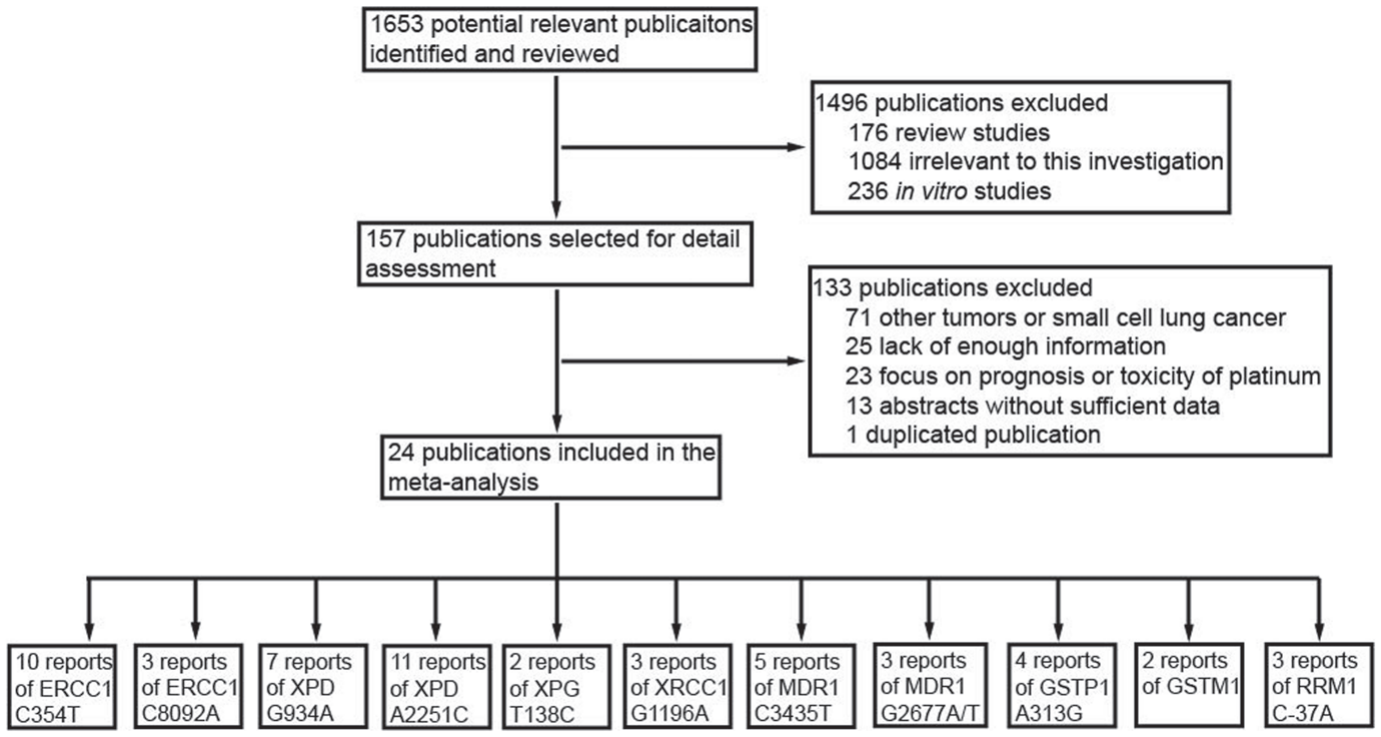
Flow chart of literature selection.

**Figure 2:**
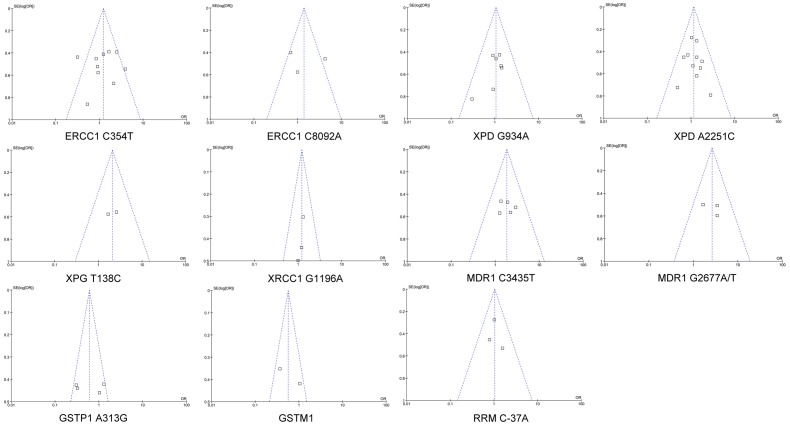
Funnel plots of SE (log [OR]) by the OR in meta-analysis . SE (log [OR]) is standard error of log [OR]. Each dot represents one article.

The contents of this meta-analysis were summarized in Table S1. Our initial electronic search retrieved 1653 publications, among which, 176 were review articles, 1084 were obviously not relevant to this investigation, and 236 were *in vitro* studies. Therefore, 157 studies were included in the next round of review. After reading the full text of these articles, we found that 71 focused on other tumors, 25 didn’t provide enough information, 19 focused on disease prognosis, 4 focused on drug side effect, 13 were conference abstracts without sufficient data, and 1 was duplicated publication. Finally, 24 publications met the inclusion criteria with enough data to be extracted (Figure 1 and S7). These publications included 11 polymorphisms in 8 genes (Table 1). Visual inspection of the funnel plots of all meta-analysis revealed a symmetrical inverted V shape (Figure 2). Begg’s test and Egger’s test also didn’t suggest evidence of publication bias. Thus, there was no publication bias for all meta-analysis in the present study. The characteristics of these studies were summarized in Table 2.

**Table 1:** Polymorphisms involved in this study and the response rate in different alleles.

Genes	Polymorphisms	NCBI SNP ID	Allele	Responders	Non-responders	References
ERCC1	C354T	rs11615	C	210	285	[6]–[8], [22], [59]–[64]
	(Asn118Asn)		T	199	308	
	C8092A	rs3212986	C	85	87	[9], [22], [65]
			A	85	93	
XPD	G934A	rs1799793	G	114	174	[7]–[9], [59], [63], [66]–[68]
	(Asp312Asn)		A	93	181	
	A2251C	rs13181	A	259	528	[7]–[9], [59], [63], [65]–[70]
	(Lys751Gln)		C	172	372	
XPG	T138C	rs1047768	C	13	20	[23], [24]
	(His46His)		T	41	131	
XRCC1	G1196A	rs25487	G	60	110	[9], [23], [68], [69]
	(Arg399Gln)		A	71	152	
MDR1	C3435T	rs1045642	C	49	46	[8], [32], [60], [63], [71]
	(Ile1145Ile)		T	88	141	
	G2677T/A	rs2032582	G	31	22	[32], [60], [71]
	(Ala893Ser/Thr)		T/A	44	67	
GSTP1	A313G	rs1695	A	64	181	[9]–[11], [72]
	(Ile105Val)		G	67	113	
GSTM1	Deletion	NR	presence	31	63	[9], [62]
			deletion	38	40	
RRM1	C-37A	NR	C	87	123	[8], [36], [63]
			A	65	92	

NR: not reported.

**Table 2:** Characteristics of studies involved in the meta-analysis.

Authors	Year	Ethnicity (Country)	Number of patients	Disease stage	Genotyping method	Chemotherapeutic drugs	Genes and polymorphism	Reference
Camps *et al.*	2003	Caucasian (Spain)	38	IIIB-IV	Direct sequencing	Cisplatin/Gemcitabine	XPD: G934A XPD: A2251C	[67]
Isla *et al.*	2004	Caucasian (Spain)	62	IIIB-IV	TaqMan genotyping assay	Cisplatin/Docetaxel	ERCC1: C354T MDR1: C3435T RRM1: A-37C XPD: G934A XPD: A2251C	[8]
Ryu *et al.*	2004	Asian (Korea)	108	IIIB-IV	SNaPShot assay	Platinum-based chemotherapy	ERCC1: C354T XPD: G934A XPD: A2251C	[59]
Booten *et al.*	2006	Caucasian (UK)	89	III-IV	Direct sequencing	Platinum-based chemotherapy	GSTP1:A313G	[11]
Booton *et al.*	2006	Caucasian (UK)	89	III-IV	PCR-RFLP Direct sequencing	Platinum-based chemotherapy	XPD: G934A XPD: A2251C	[66]
Su *et al.*	2007	Asian (China)	76	IIIA-IV	TaqMan genotyping assay	Platinum-based chemotherapy	ERCC1: C354T	[6]
Giachino *et al.*	2007	Caucasian (Italy)	188	IIIA-IV	PCR-RFLP	Platinum-based chemotherapy	XRCC1: G1196A XPD: A2251C	[69]
Tibaldi *et al.*	2008	Caucasian (Italy)	65	IIIB-IV	TaqMan genotyping assay	Cisplatin/Gemcitabine	ERCC1: C354T XPD: G934A XPD: A2251C	[7]
Pan *et al.*	2008	Asian (China)	69	IIIB-IV	PCR-RFLP	Cisplatin/Vinorelbine	MDR1: C3435T MDR1: G2677A/T	[71]
Yu *et al.*	2008	Asian (China)	117	NR	Direct sequencing	Carboplatin/Etoposide	ERCC1: C354T ERCC1: C8092A	[22]
Kalikaki *et al.*	2009	Caucasian (Greece)	119	IIIA-IV	PCR-RFLP Direct sequencing	Platinum-based chemotherapy	ERCC1: C354T ERCC1: C8092A GSTP1: A313G GSTM1: deletion XPD: G934A XPD: A2251C XRCC1: G1196A	[9]
Feng *et al.*	2009	Asian (China)	214	IIB-IV	PCR-RFLP	Platinum-based chemotherapy	RRM1: A-37C	[36]
Yao *et al.*	2009	Asian (China)	102	IIIB-IV	PCR-RFLP	Platinum-based chemotherapy	XRCC1: G1196A	[68]
Sun *et al.*	2009	Asian (China)	90	IV	3-D polyacrylamide gel-based DNA microarray	Platinum-based chemotherapy	XRCC1: G1196A	[23]
Pan *et al.*	2009	Asian (China)	54	IIIB-IV	PCR-RFLP	Cisplatin/Docetaxel	MDR1: C3435T MDR1: G2677A/T	[32]
Zhou *et al.*	2010	Asian (China)	130	IIIB-IV	TaqMan genotyping assay	Platinum-based chemotherapy	ERCC1: C354T	[61]
Chen *et al.*	2010	Asian (China)	95	IIIB-IV	Ligase detection reactions (LDR)	Platinum-based chemotherapy	ERCC1: C354T MDR1: C3435T MDR1: G2677A/T	[60]
Sun *et al.*	2010	Asian (China)	113	IIIA-IV	3-D polyacrylamide gel-based DNA microarray	Platinum-based chemotherapy	GSTP1: A313G	[10]
Li *et al.*	2010	Asian (China)	115	IIIB-IV	3-D polyacrylamide gel-based DNA microarray	Platinum-based chemotherapy	ERCC1: C354T XPD: A2251C	[65]
Vinolas *et al.*	2011	Caucasian (Spain)	94	IIIB-IV	5′ nuclease allelic discrimination assay	Cisplatin/Vinorelbine	ERCC1: C354T XPD: G934A XPD: A2251C MDR1: C3435T RRM1: A-37C	[63]
Zhou *et al.*	2011	Asian (China)	111	IV	Direct sequencing	Platinum-based chemotherapy	GSTP1: A313G	[72]
Li *et al.*	2012	Asian (China)	58	NR	PCR-RFLP	Platinum-based chemotherapy	GSTM1:deletion	[62]
Chen *et al.*	2012	Asian (China)	355	IIIB-IV	TaqMan genotyping assay	Platinum-based chemotherapy	XPD: A2251C	[70]
Cheng *et al.*	2012	Asian (China)	142	IIIB-IV	Direct sequencing	Platinum-based chemotherapy	ERCC1: C354T	[64]

NR: not reported.

### 2. Nucleotide Excision Repair (NER) Pathway

DNA repair was one of the most important pathways involved in platinum response. Therefore, polymorphisms of genes in DNA repair pathway may affect patients’ response to platinum chemotherapy. There were four major DNA repair pathways: nucleotide excision repair (NER), base excision repair (BER), double-strand break repair (DSB) and mismatch repair (MMR) [20]. NER was the major repair system for removal of platinum-caused DNA lesions [20], [21]. Thus it was closely related with platinum response. Our meta-analysis contained 3 genes in NER pathway: ERCC1, xeroderma pigmentosum group D (XPD) and xeroderma pigmentosum group G (XPG).

#### 2.1 ERCC1

**Figure 3:**
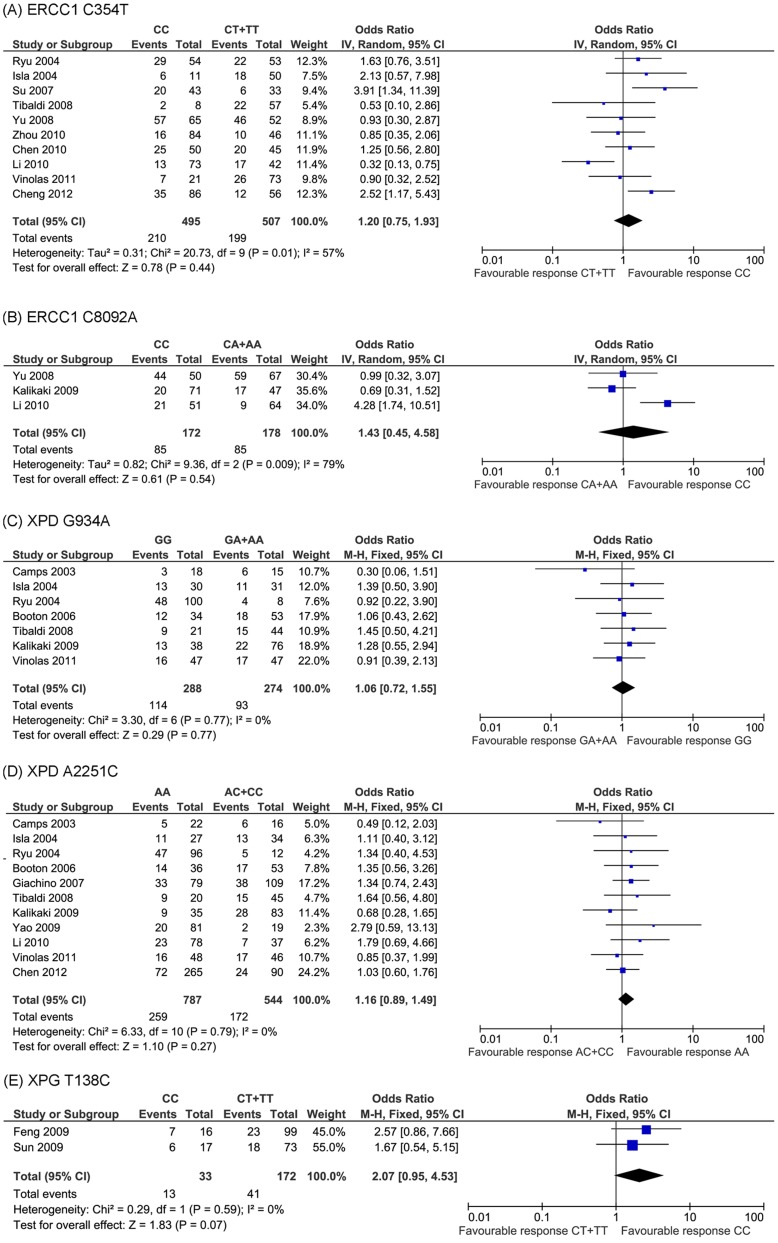
Meta-analysis of association polymorphisms in NER DNA repair pathway genes and platinum-based chemotherapy in NSCLC patients. No significant association was found for these polymorphisms.

The most extensively studied polymorphism was ERCC1 C354T. Ten studies examined the association between this polymorphism and platinum-based drug response in NSCLC patients. We conducted a meta-analysis on these studies, which included 1002 subjects (495 CC genotype and 507 CT+TT genotype carriers). Because there was heterogeneity across the studies (*P* = 0.01, I^2^ = 57%), we chose random-effect model. Pooled data from these investigations showed 42.4% and 39.3% overall response rate in CC genotype and CT+TT genotype group, respectively. No significant relationship was detected between ERCC1 C354T and drug response (OR = 1.20, 95% CI: 0.75−1.93, *P* = 0.44) (Figure 3A). We further analyzed their relationship stratified by ethnic populations. The result showed that ERCC1 C354T was not correlated with drug response in either Asian or Caucasian population (Figure S1).

C8092A was another polymorphism of ERCC1 that was included in this study [9], [22]. Since heterogeneity was also identified among the three studies (*P* = 0.009, I^2^ = 79%), random-effect model was selected. Again, no significant association between ERCC1 C8092A and drug response was detected (OR = 1.43, 95% CI: 0.45−4.58, *P* = 0.54) (Figure 3B).

#### 2.2 XPD

Another intensively investigated gene in the NER pathway was XPD. Two polymorphisms (G934A and A2251C) were widely studied. Meta-analysis of G934A and A2251C included 7 and 11 studies, respectively. No heterogeneity was found across these studies (*P* = 0.77, I^2^ = 0% and *P* = 0.79, I^2^ = 0% for G934A and A2251C, respectively), thus we chose fixed-effect model. For XPD G934A, pooled data contained 562 patients. Response rates for the GG genotype carriers and GA+AA genotype carriers were 39.6% and 33.9%, respectively. However, the OR (1.06) and 95% CI (0.72−1.55) values indicated that there was no significant correlation between this polymorphism and platinum-based chemotherapy (*P* = 0.77) (Figure 3C). In terms of XPD A2251C, 11 studies included 1331 individuals in total. The response rate for AA genotype was 32.9%, while that for AC+CC genotype was 31.6%. No significant association was found between this polymorphism and drug response (OR = 1.16, 95% CI: 0.89−1.49, *P* = 0.27) (Figure 3D). Analysis stratified by different populations was further investigated, with no significant correlation detected for polymorphism in either Asian or Caucasian population (Figures S3 and S4). Taken together, neither polymorphism of XPD was significantly related with platinum-based chemotherapy in NSCLC patients.

#### 2.3 XPG

Another genetic polymorphism in the XP family involved in this study was XPG T138C. Sun *et al.* showed that it was significantly associated with platinum-based chemotherapy (*P* = 0.047) [23] while, another study didn’t find significant correlation between this SNP and chemotherapeutic drug response (OR = 2.57, 95% CI: 0.86−7.66, *P* = 0.083) [24]. T138C was a synonymous mutation. Our meta-analysis contained these two contradictory investigations, and no heterogeneity was found across these two studies (*P* = 0.59, I^2^ = 0%). As a result, no significant correlation was found between this polymorphism and drug response (OR = 2.07, 95% CI: 0.95−4.53, *P* = 0.07) (Figure 3E).

### 3. Other Pathways

Although NER was the most important pathway for platinum drug resistance, other genetic polymorphisms were also widely studied, including other DNA repair pathways, detoxification system and drug transporters.

#### 3.1 X-Ray Cross-Complementing Group 1 (XRCC1)

**Figure 4:**
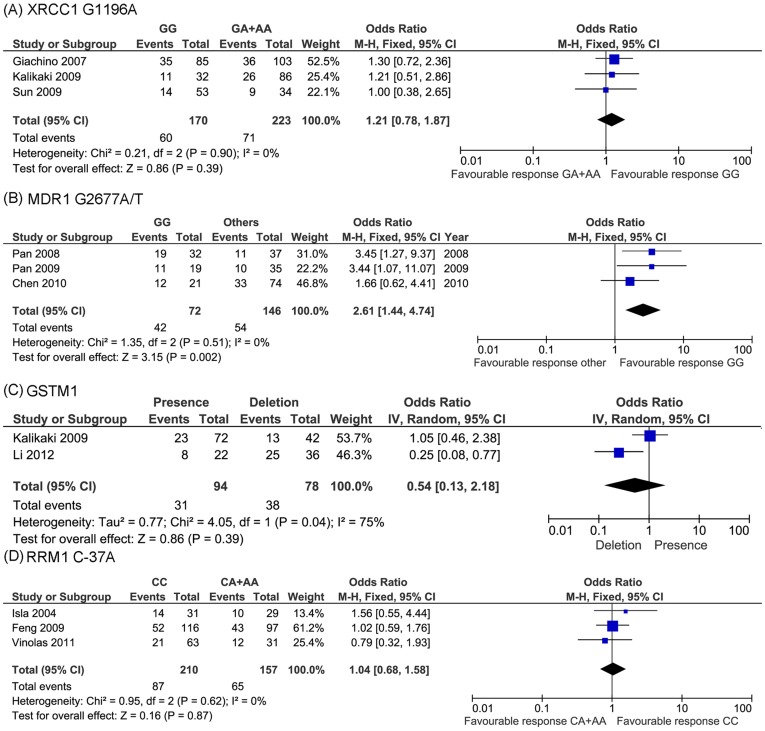
Meta-analysis of association between other pathways gene polymorphisms and platinum-based chemotherapy in NSCLC patients. No significant association was found for XRCC1 G1196A (A), GSTM1 (C) and RRM C-37A (D), while significant correlation was identified for MDR1 G2677A/T (*P* = 0.002) (B).

Another extensively studied DNA repair gene is XRCC1, which belongs to BER pathway. In this meta-analysis, three publications investigated G1196A of XRCC1. No heterogeneity was found across the three studies (*P* = 0.90, I^2^ = 0%). Thus fixed-effect model was selected. The pooled data showed that no significant association was found between this polymorphism and drug response (OR = 1.21, 95% CI: 0.78−1.87, *P* = 0.39) (Figure 4A).

#### 3.2 Multidrug resistance 1 (MDR1)

The decreased accumulation of drug in the cell has been proved to be a common mechanism of drug resistance [25]. There were two major causes of reduced intracellular drug accumulation: decreased drug influx and increased drug efflux. In terms of platinum resistance, polymorphisms of MDR1 were extensively studied.

**Figure 5:**
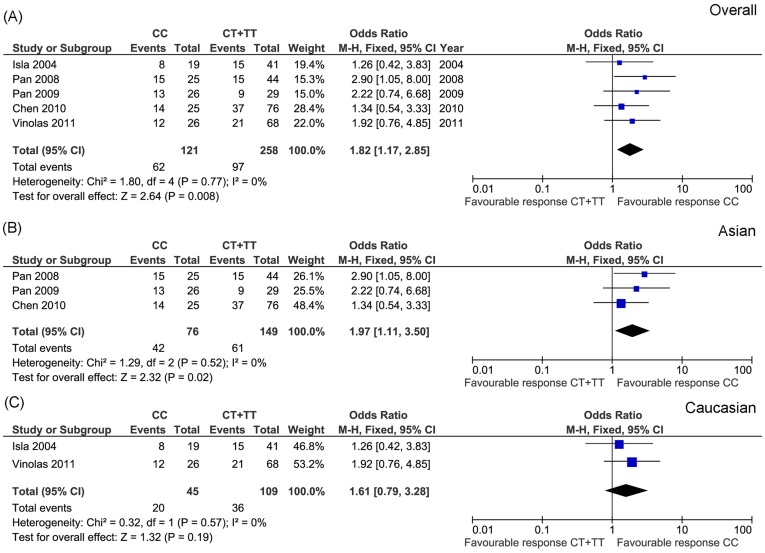
Meta-analysis of association between MDR1 C3435T and platinum-based chemotherapy in overall (A), Asian (B) and Caucasian (C) NSCLC patients. Significant association was identified in overall (*P* = 0.008) and Asian (*P* = 0.02) populations.

Five studies investigated MDR1 C3435T, including a total of 379 patients, the response rates in the CC and CT+TT genotype group were 51.2% and 37.6%, respectively. No heterogeneity across the studies was found (*P* = 0.77, I^2^ = 0%). We thus selected fixed-effect model. The result showed that this polymorphism was significantly correlated with drug response (OR = 1.82, 95% CI: 1.17−2.85, *P* = 0.008) (Figure 5A). CC genotype carrier showed significant increased drug response. Considering that ethnic differences between populations may contribute to the drug response and our included five studies comprise two different ethnic populations, we further conducted separate analyses in Asian and Caucasian population, respectively. It was interesting to note that MDR1 C3435T was only significantly correlated with platinum response in Asian population (*P* = 0.02) (Figure 5B). No association was detected in Caucasian population (*P* = 0.19) (Figure 5C).

Another MDR1 polymorphism involved in this meta-analysis was G2677A/T. Heterogeneity test showed that fixed-effect model should be selected (*P* = 0.51, I^2^ = 0%). It was interesting to found that this SNP was also significantly associated with platinum response (OR = 2.61, 95%CI: 1.44−4.74, *P* = 0.002), patients with GG genotype had better response to platinum based chemotherapy (Figure 4B). Furthermore, it is noteworthy that all studied included for this SNP were conducted in the Asian population. Thus, taken together, MDR1 polymorphisms were correlated with NSCLC patients’ platinum-based chemotherapy in Asian population.

#### 3.3 Glutathione S-transferase P1 (GSTP1)

Once platinum enters the cells, it could be conjugated by glutathione (GSH). The formed complex reduces the toxicity of platinum and can be more easily transported out of the cells. The conjugation is determined by the cellular GSH level. Therefore, some GSH synthesis enzymes are correlated with platinum response. One of the most important enzymes is glutathione S-transferase (GST), which includes several members such as GSTP1, GSTM1 and GSTT1.

**Figure 6:**
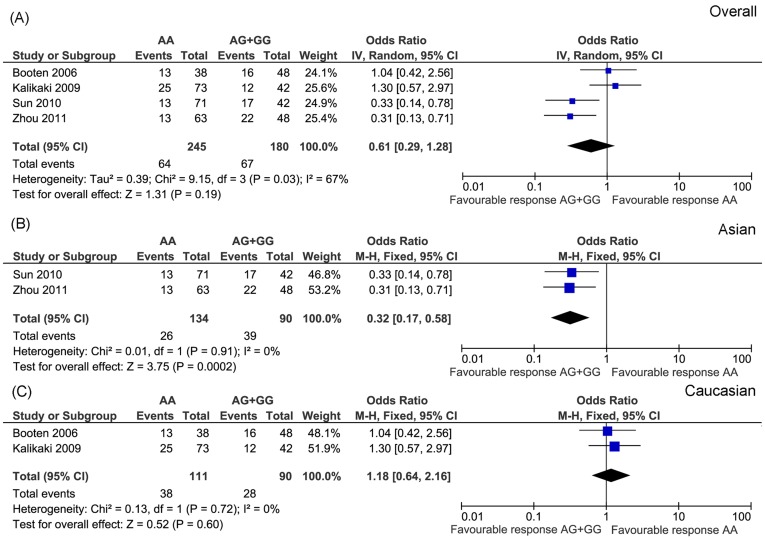
Meta-analysis of association between GSTP1 A313G and platinum-based chemotherapy in overall (A), Asian (B) and Caucasian (C) NSCLC patients. Significant association was identified in the Asian (*P* = 0.0002) populations.

GSTP1 A313G was the most widely studied polymorphism. We included four publications and conducted a meta-analysis. These studies included 425 patients. Response rates of the AA and AG+GG genotype group were 26.1% and 37.2%, respectively. Because heterogeneity was detected across these studies (*P* = 0.03, I^2^ = 67%), we selected random-effect model. The result showed no association between GSTP1 A313G and platinum-based chemotherapy (OR = 0.61, 95% CI: 0.29−1.28, *P* = 0.19) (Figure 6A). To avoid the additional heterogeneity introduced by different ethnic population, we further analyzed the correlation stratified by different populations. As showed in Figure 6 B and C, GSTP1 A313G was only significantly associated with drug response in Asian population.

One deletion polymorphism of GSTM1 was also investigated in this study, heterogeneity was detected across involved two studies (*P* = 0.04, I^2^ = 75%), random-effect model was used to evaluate correlation between GSTM1 polymorphism and drug response. However, no significant association was detected (OR = 0.54, 95% CI: 0.13−2.18, *P* = 0.39) (Figure 4C).

#### 3.4 Ribonucleotide reductase M1 (RRM1)

The last enrolled gene was RRM1. Three studies investigated the polymorphism of C-37A. No heterogeneity was detected across the studies (*P* = 0.62, I^2^ = 0%). Thus fixed-effect model was selected. We didn’t find significant correlation between this SNP and drug response (OR = 1.04, 95% CI: 0.68−1.58, *P* = 0.87) (Figure 4D).

## Discussion

In this study, we investigated the phamacogenetics of platinum-based chemotherapy in NSCLC patients. We conducted meta-analysis for 11 polymorphisms in 8 genes. The results showed that MDR1 C3435T, G2677A/T and GSTP1 A313G were significantly correlated with platinum-based chemotherapy in the Asian NSCLC patients.

Platinum is an effective chemotherapeutic drug for lung cancer patients. However, the mechanisms for individual difference on drug response remain unknown. Gene polymorphisms have been proved to play an important role in the drug therapeutic efficacy [5], [25]–[27]. Therefore, researchers focused on the polymorphisms of the genes in the platinum pathways [12]. In the present study, it is interesting that two polymorphisms of MDR1 were significantly correlated with platinum response. MDR1 encodes P-glycoprotein (P-gp), which is responsible for transporting a broad spectrum of drugs out of cells [28]. It was widely reported to be implicated in platinum resistance due to its activity of drug efflux [29]. Thus it is not surprising that MDR1 polymorphisms were associated with platinum response. However, the mechanisms of their correlation remain unclear. One proposal is that these polymorphisms affect P-gp protein expression or function which, in turn, alter drug efflux activity. A number of publications reported the association between MDR1 C3435T and G2677A/T and P-gp expression or function. The mutated MDR1 had altered P-gp expression level or aberrant protein function [30], [31]. It was previously reported that C3435T and G2677A/T were in linkage disequilibrium (LD) with each other [32]. Thus their haplotype may also have significant contribution to platinum response. However, existing literatures do not provide enough data for a meta-analysis for this hypothesis. Therefore, their correlation awaits further investigations. Another polymorphism found to be significantly correlated with drug response was GSTP1 A313G, which is a non-synonymous SNP located in exon 5 [33]. GSTs are phase II metabolic enzymes involved in the platinum detoxification mediated by glutathione (GSH) conjugation [34]. GSTP1 is one of the major isoforms of GST and its activity level is associated with platinum detoxification and subsequent drug response. Previous study showed that GSTP1 A313G resulted in reduced glutathione conjugating ability [33]. Thus, patients harboring this polymorphism may have decreased platinum detoxification capacity, which is consistent with our result that mutated genotype carriers were more sensitive to platinum-based chemotherapy.

Except for drug transporters and detoxification, DNA repair was also one of the classical platinum resistance mechanisms [34], We proposed that genetic polymorphisms in DNA repair pathways were correlated with drug response prior to this study. However, we failed to find the significant correlation between genetic polymorphisms in any DNA repair pathway and platinum chemotherapy response in this study. To further verify this result, we examined more DNA repair pathway polymorphisms, which were not included in this study because they didn’t meet our including criteria. Among these, ERCC1 T262G [22], hMSH2 IVS12-6T>C [35], RRM1 C-524T [36], XPA A23G [24] and XRCC1 T194C [23] were claimed to be significantly associated with drug response by other authors. However, after careful examination on these studies, we found several problems in these studies. For example, adjusted OR values were not calculated for RRM1 C-524T. Besides, the results of hMSH2 IVS12-6T>C and XRCC1 T194C were inconsistent between different genotypes. We did not find similar problems in the analyses of ERCC1 T262G and XPA A23G. However, due to the small sample size, we think these results need to be further validated. Taken both our and these studies together, the association between genetic polymorphisms in DNA repair pathway and platinum-based chemotherapy in NSCLC is not supported by experimental evidence.

Considering the importance of these genes in platinum resistance, especially for NER DNA repair pathway genes, it is surprising that no correlation was detected. We think one possibility is that cancer genetics is different from somatic genetics. Although a number of studies showed that polymorphisms in these genes affect their expression or function [37], [38], they may have no effect on cancer patients’ chemotherapeutic efficiency. We understand that many other cancer genetic factors also affect the therapeutic efficiency of platinum. Thus, the polymorphisms in DNA repair pathway genes may not play a central role in platinum drug response in NSCLC. This is supported by a large sample size study conducted by Shiraishi *et al* [39], who genotyped 30 SNPs in 27 DNA repair genes in 640 NSCLC patients. Some of the polymorphisms were also investigated in the present study such as XPD A2251C and XRCC1 G1196A. Their results were in agreement with ours in that, most of polymorphisms in the DNA repair pathway genes were not significantly involved in platinum drug response. Our results are also supported by several recent large genome wide association studies (GWAS) in NSCLC [40]–[42]. Wu *et al.* conducted a GWAS (including 307,260 SNPs) in a total of 1062 NSCLC patients, they identified that the rs1878022 in the chemokine-like receptor 1 (CMKLR1) was statistically associated with poor overall survival [41]. No polymorphisms in the DNA repair pathway genes were identified. Another GWAS study performed by Yang group also didn’t identify the correlation between DNA repair gene polymorphisms and patients overall survival in NSCLC [42], either. They explored 894 SNPs in 70 genes in four pathways related to chemotherapy drug actions, including GSH, DNA repair, cell cycle and EGFR pathways. However, this 1076 patients study concluded that only genetic variations in EGFR and glutathione pathways were associated with overall survival in NSCLC patients receiving platinum based chemotherapy. Although the endpoints in these studies were overall survival, considering the close association between drug response and survival, they may also provide some clues for the correlation between these SNPs and drug response. Taken together, compared with DNA repair pathway genetic polymorphisms, other gene polymorphisms may have more contribution to chemotherapy response in NSCLC patients receiving platinum-based treatment.

Lung cancer is a heterogeneous disease. Thus pathological and ethnic difference may affect drug response. To clarify this concern, we analyzed the correlation between genetic polymorphisms and drug response stratified by different ethnic populations (Figure 5, 6 and S1, S2, S3, S4, S5, and S6). Although most of the separately analyzed results were consistent with overall population, two polymorphisms (MDR1 C3435T and GSTP1 A313G) showed ethnic difference. Both polymorphisms were only significantly associated with drug response in Asian population, not in Caucasian population (Figure 5 and 6). This result indicates that the outcomes of platinum-based chemotherapy differ between Asian and Caucasian population. Thus, ethnic factor should be considered if personalized chemotherapy treatment is conducted in the future.

Pathology is another factor to be considered when pooled data was analyzed, because NSCLC includes adenocarcinoma and squamous cell carcinoma. Although both types of tumor are treated with the same regimen, the outcomes may be completely different. However, studies included in this meta-analysis didn’t provide the data of response rate stratified by both polymorphisms and pathological types. Thus it is impossible to do the meta-analysis separately on adenocarcinoma and squamous cell carcinoma based on existing studies. This is one of the limitations of our study. To address this issue, future studies are needed when enough data are available.

We also identified some flaws in existing studies. One common flaw is that the authors didn’t perform comprehensive analysis. As our result indicated, a single polymorphism has little contribution to chemotherapy efficacy. Thus comprehensive analysis was needed. One restriction for the past comprehensive study is shortage of high-throughput genotyping methods. With the advancement of technology, more and more methods have been developed for performing high-throughput genotyping, which can be used in the future study. For example, in our included studies, one group used 3D polyacrylamide gel-based DNA microarray to determine genotypes [10], [23], [24], [35]. This is a powerful high-throughput genotyping assay, which is frequently utilized by this group. As gene sequencing technology develops rapidly, another available method is genome-wide approach. One group identified some variants that were related with platinum response using this method [43]–[46]. However, all these studies are *in vitro* investigations and the results need to be verified in the patients. On the other hand, GWAS have been successfully used in disease susceptibility and pharmacogenetic research [47]–[50]. Thus, we believe GWAS is a powerful tool to investigate the pharmacogenetics of platinum-based chemotherapy in the future. Another problem is inadequate sample size. The sample sizes of involved studies were from 33 to 355, and none of these studies did a sample size calculation. In some situation, especially for the low frequency polymorphisms, small sample size cannot generate solid conclusion. Furthermore, as described previously, some studies didn’t calculate adjusted OR [36], [51]. They just used χ^2^ test to assess the distribution of different genotypes in responders and non-responders. Thus, these studies can’t exclude the effect of age, sex, smoking habit, tumor histology, and tumor stages. To make the result more reliable, future studies should avoid these pitfalls.

The cellular process of platinum is complicated, which includes a lot of pathways. However, existing studies didn’t cover all platinum pathways. Therefore, some most important polymorphisms affecting platinum chemotherapy may still be elusive. For instance, compared with efflux, drug influx is also crucial for platinum accumulation in the tumor cells. Copper transporter-1 (CTR1) and ATPases ATP7 have a substantial role in platinum influx [52]–[54]. However, no polymorphisms of these genes were investigated. Other important pathways that didn’t attract attention include DNA damage signal transduction pathways. These pathways are, but not limited to, AKT, c-ABL, p53, MAPK/JNK/ERK and p38 MAPK [55]. We didn’t find publications investigating polymorphisms of genes in these pathways. Therefore, we propose that another possible direction in the future is to investigate genes in these pathways.

Prior to this study, another two groups performed meta-analysis to detect the correlation of ERCC1 and MDR1 polymorphisms and platinum-based chemotherapies in advanced NSCLC [56], [57]. Our results are in agreement with these two studies. However, a study conducted by Wei *et al.* had different results. They investigated the association between ERCC1 and XPD polymorphisms and platinum-based chemotherapy in advanced NSCLC patients [58]. They studied ERCC1 C354T, XPD G934A and A2251C, and found that ERCC1 C354T was significantly correlated with drug response in patients treated with platinum-based chemotherapy. Apparently, their result is inconsistent with our study. We think that the discrepancy comes from the difference in enrolled studies. Our meta-analysis included six more studies, five of which are published after Wei’s meta-analysis. In the other one, data were obtained directly from the author. Besides, our investigation didn’t include two studies, which were in Wei’s investigation. One study was not published in English, and the other had errors in genotype classification. As a result, our meta-analysis included 1002 patients, while Wei’s study included 556 patients. Therefore, the difference in patients pool possibly caused inconsistency in results.

In conclusion, we identified that MDR1 C3435T, G2677A/T and GSTP1 A313G were significantly correlated with platinum-based chemotherapy in Asian NSCLC patients. These polymorphisms should be considered for personalized chemotherapy treatment for NSCLC patients in Asian population in the future.

## Supporting Information

Figure S1 Meta-analysis of association between ERCC1 C354T and platinum-based chemotherapy in NSCLC patients stratified by ethnic population. No significant association was found in either Asian (A) or Caucasian (B) population.
(TIF)

Figure S2 Meta-analysis of association between ERCC1 C8092A and platinum-based chemotherapy in Caucasian NSCLC patients. No significant association was identified.
(TIF)

Figure S3 Meta-analysis of association between XPD G934A and platinum-based chemotherapy in Caucasian NSCLC patients. No significant association was detected.
(TIF)

Figure S4 Meta-analysis of association between XPD A2251C and platinum-based chemotherapy in NSCLC patients stratified by ethnic population. No significant association was found in either Asian (A) or Caucasian (B) populations.
(TIF)

Figure S5 Meta-analysis of association between XRCC1 G1196A and platinum-based chemotherapy in Caucasian NSCLC patients. No significant association was found.
(TIF)

Figure S6 Meta-analysis of association between RRM1 C-37A and platinum-based chemotherapy in Caucasian NSCLC patients. No significant association was detected.
(TIF)

Figure S7 PRISMA flow diagram
(DOC)

Table S1 PRISMA checklist
(DOC)
