# Supplementary figures and images for: Meta-Analysis on Pharmacogenetics of Platinum-Based Chemotherapy in Non Small Cell Lung Cancer (NSCLC) Patients
Source: PLoS One. 2012 Jun 26;7(6):e38150. doi: 10.1371/journal.pone.0038150 (PMC3383686; doi:10.1371/journal.pone.0038150)

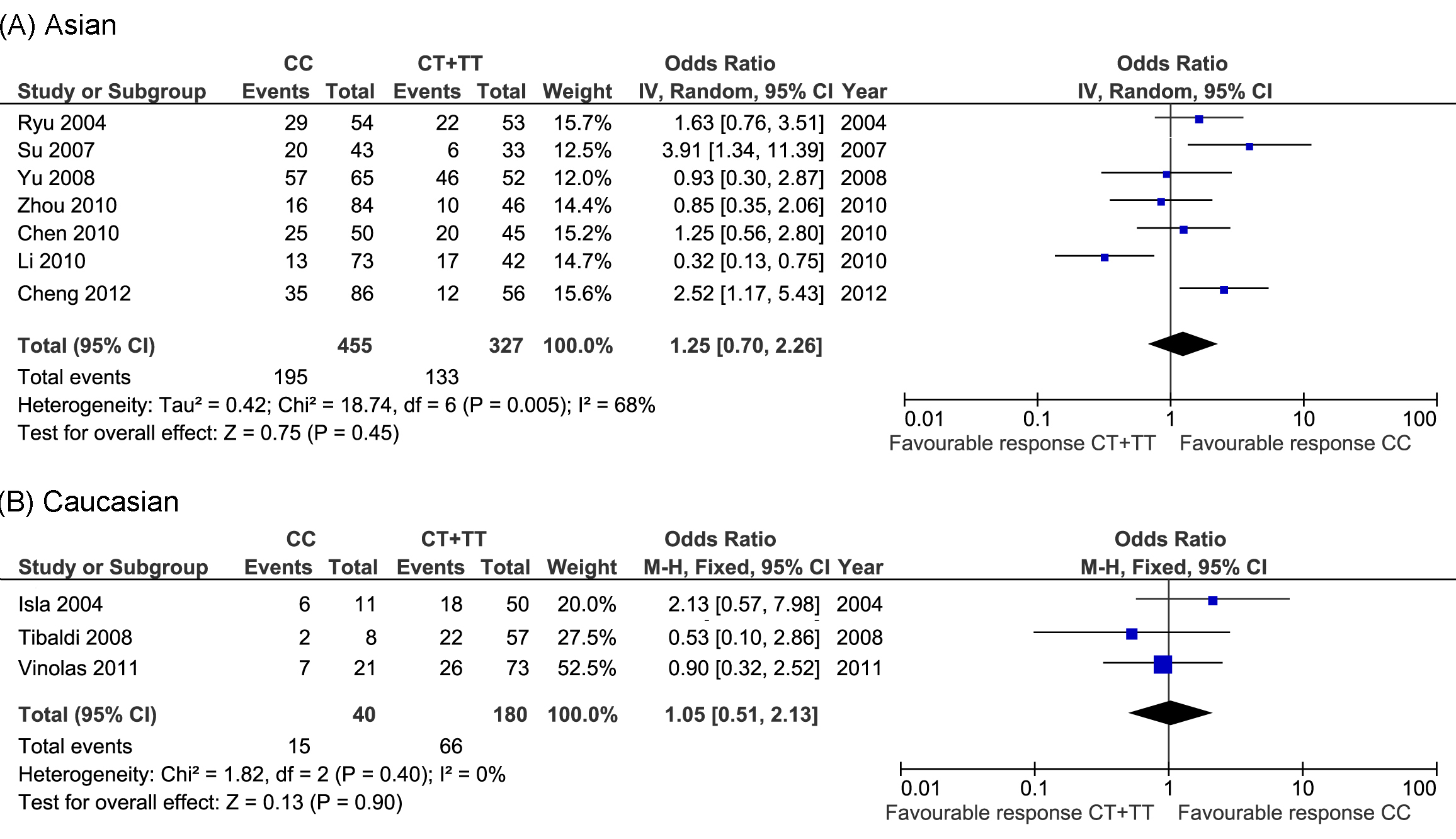

Supplement: Figure S1 — Meta-analysis of association between ERCC1 C354T and platinum-based chemotherapy in NSCLC patients stratified by ethnic population. No significant association was found in either Asian (A) or Caucasian (B) population. (TIF) [file pone.0038150.s001.tif]

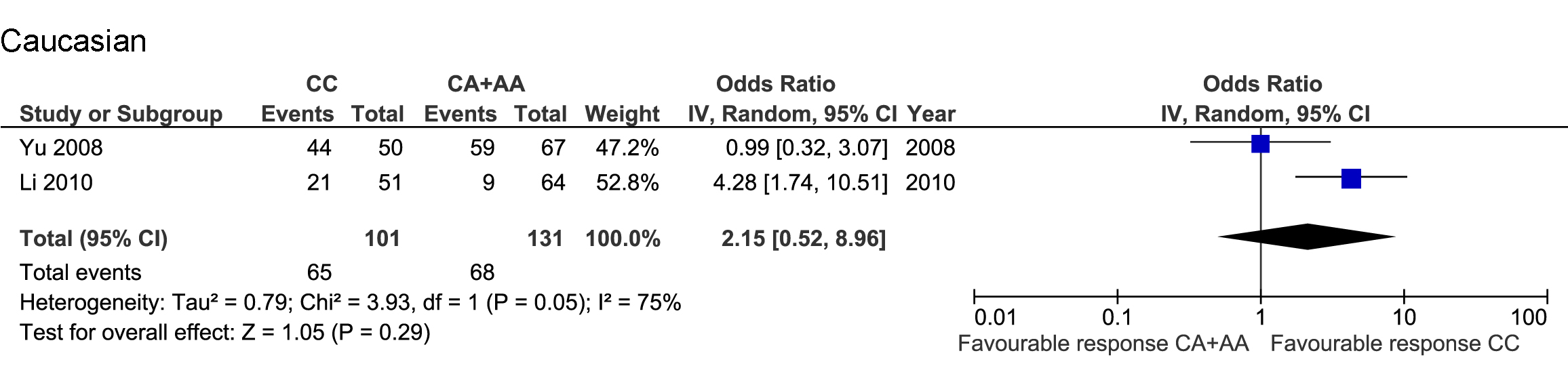

Supplement: Figure S2 — Meta-analysis of association between ERCC1 C8092A and platinum-based chemotherapy in Caucasian NSCLC patients. No significant association was identified. (TIF) [file pone.0038150.s002.tif]

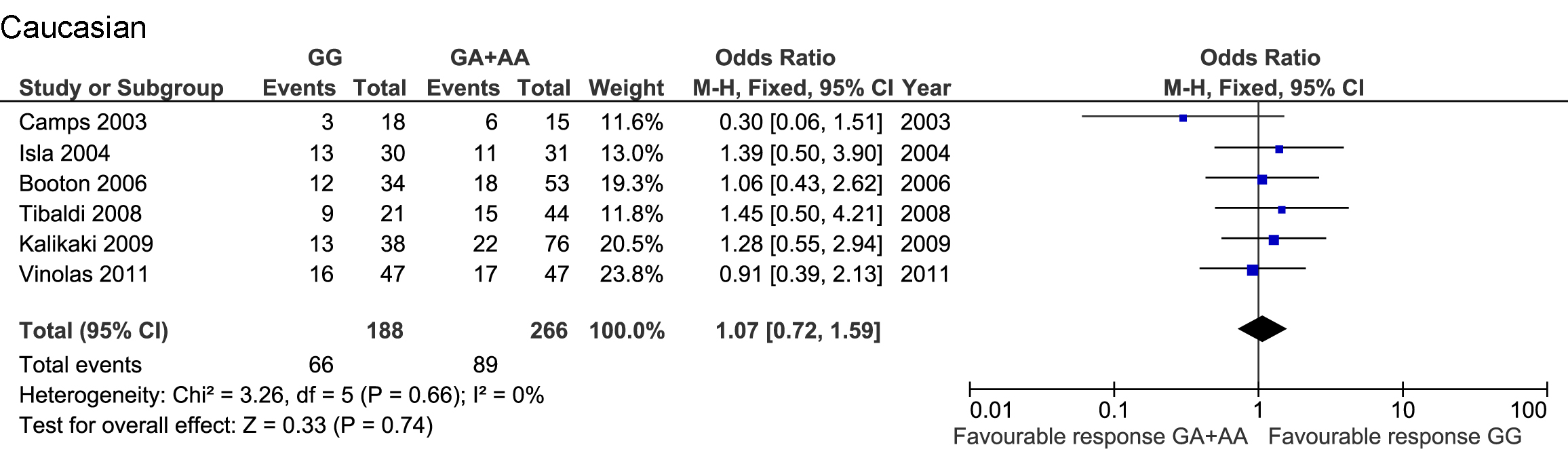

Supplement: Figure S3 — Meta-analysis of association between XPD G934A and platinum-based chemotherapy in Caucasian NSCLC patients. No significant association was detected. (TIF) [file pone.0038150.s003.tif]

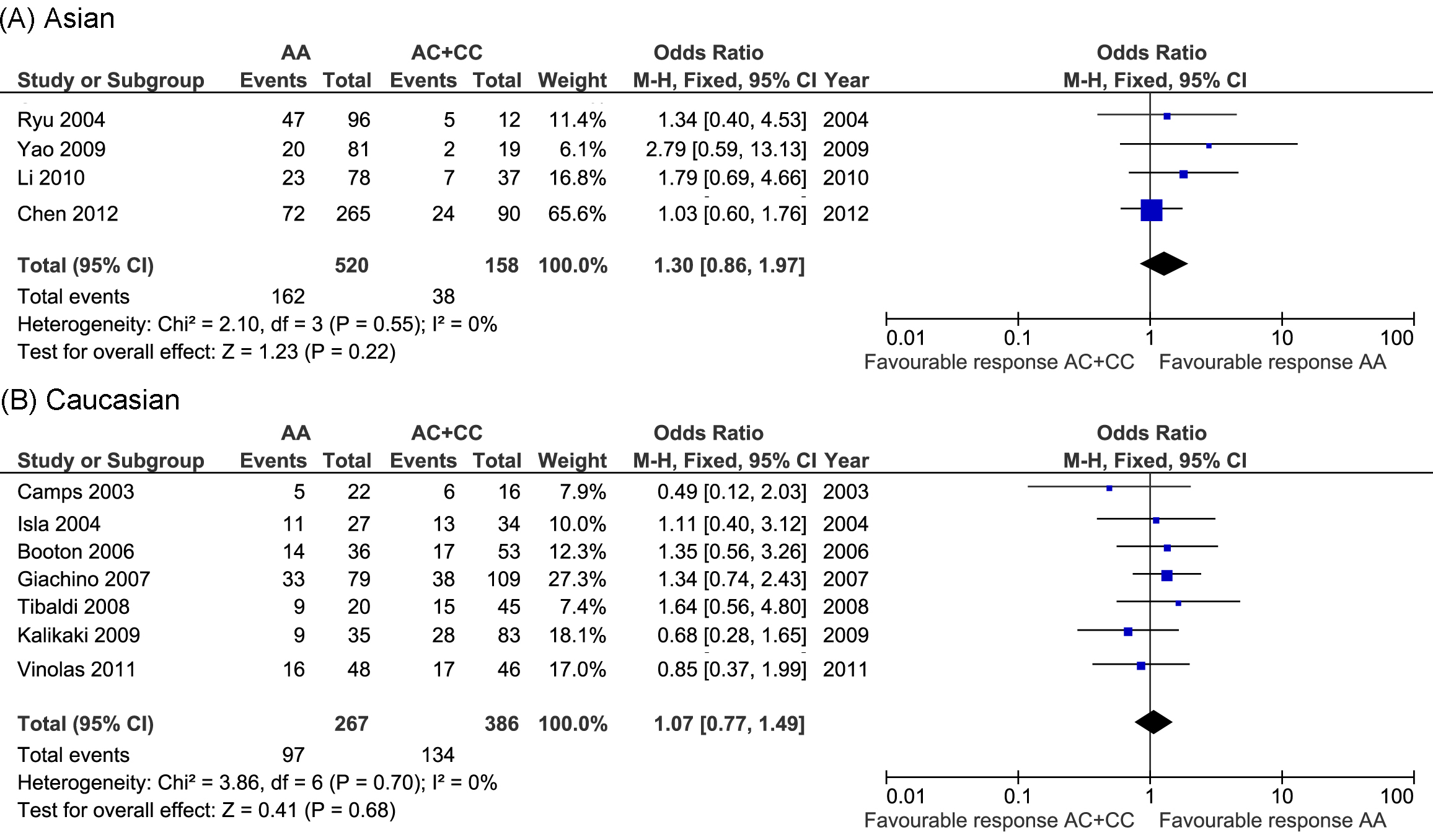

Supplement: Figure S4 — Meta-analysis of association between XPD A2251C and platinum-based chemotherapy in NSCLC patients stratified by ethnic population. No significant association was found in either Asian (A) or Caucasian (B) populations. (TIF) [file pone.0038150.s004.tif]

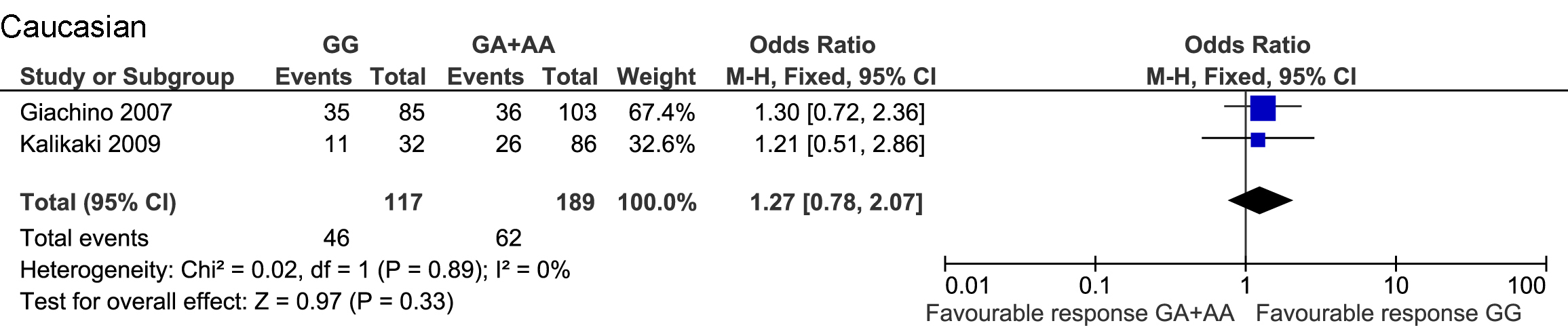

Supplement: Figure S5 — Meta-analysis of association between XRCC1 G1196A and platinum-based chemotherapy in Caucasian NSCLC patients. No significant association was found. (TIF) [file pone.0038150.s005.tif]

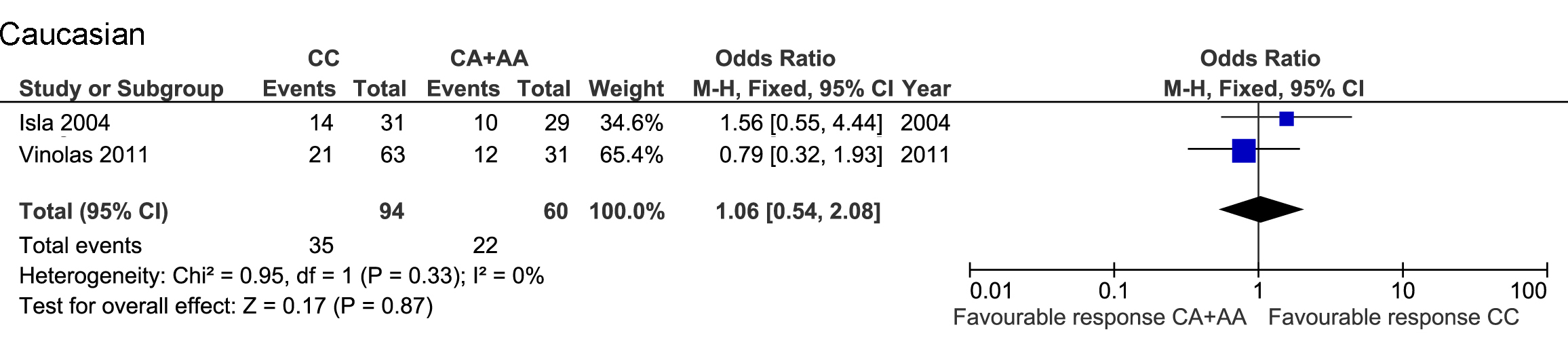

Supplement: Figure S6 — Meta-analysis of association between RRM1 C-37A and platinum-based chemotherapy in Caucasian NSCLC patients. No significant association was detected. (TIF) [file pone.0038150.s006.tif]
