# Supplementary material for: Meta-Analysis on Pharmacogenetics of Platinum-Based Chemotherapy in Non Small Cell Lung Cancer (NSCLC) Patients
Source: PLoS One. 2012 Jun 26;7(6):e38150. doi: 10.1371/journal.pone.0038150 (PMC3383686; doi:10.1371/journal.pone.0038150)
Supplement: Figure S7 — PRISMA flow diagram (DOC) [file pone.0038150.s007.doc]

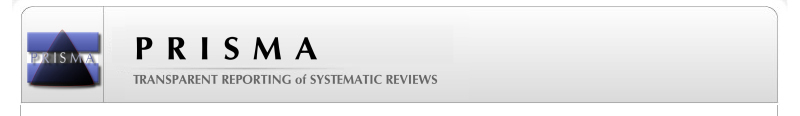
**PRISMA 2009 Flow Diagram**

**Screening**

**Included**

**Eligibility**

**Identification**

Records identified through database searching
(n =1655 )

Additional records identified through other sources
(n = 0 )

Records after duplicates removed
(n = 1653 )

Records screened
(n = 1653 )

Records excluded
(n = 1496 )

Full-text articles assessed for eligibility
(n =157 )

Full-text articles excluded, with reasons
(n = 133 )

Studies included in qualitative synthesis
(n =24 )

Studies included in quantitative synthesis (meta-analysis)
(n = 24 )
